# An In Vivo Assay of Synaptic Function Mediating Human Cognition

**DOI:** 10.1016/j.cub.2011.06.053

**Published:** 2011-08-09

**Authors:** Rosalyn J. Moran, Mkael Symmonds, Klaas E. Stephan, Karl J. Friston, Raymond J. Dolan

**Affiliations:** 1 Wellcome Trust Centre for Neuroimaging, Institute of Neurology, University College London, London WC1N 3BG, UK; 2 Laboratory for Social and Neural Systems Research, Department of Economics, University of Zurich, 8006 Zurich, Switzerland

## Abstract

The contribution of dopamine to working memory has been studied extensively [1–3]. Here, we exploited its well characterized effects [1–3] to validate a novel human in vivo assay of ongoing synaptic [4, 5] processing. We obtained magnetoencephalographic (MEG) measurements from subjects performing a working memory (WM) task during a within-subject, placebo-controlled, pharmacological (dopaminergic) challenge. By applying dynamic causal modeling (DCM), a Bayesian technique for neuronal system identification [6], to MEG signals from prefrontal cortex, we demonstrate that it is possible to infer synaptic signaling by specific ion channels in behaving humans. Dopamine-induced enhancement of WM performance was accompanied by significant changes in MEG signal power, and a DCM assay disclosed related changes in synaptic signaling. By estimating the contribution of ionotropic receptors (AMPA, NMDA, and GABA_A_) to the observed spectral response, we demonstrate changes in their function commensurate with the synaptic effects of dopamine. The validity of our model is reinforced by a striking quantitative effect on NMDA and AMPA receptor signaling that predicted behavioral improvement over subjects. Our results provide a proof-of-principle demonstration of a novel framework for inferring, noninvasively, neuromodulatory influences on ion channel signaling via specific ionotropic receptors, providing a window on the hidden synaptic events mediating discrete psychological processes in humans.

## Highlights

► We present a DCM capable of assaying neurotransmitter function during human cognition ► We demonstrate this using dopaminergic modulation of working memory and MEG ► We find changes in ionotropic receptors commensurate with dopaminergic enhancement ► We uncover quantitative effects that can predict individual behavioral improvements

## Results

In this study, we tested whether dynamic causal modeling (DCM) could recover changes in neurotransmission induced experimentally by the actions of the catecholamine dopamine. This inference about cellular processes from measured magnetoencephalographic (MEG) data aims to provide a demonstration of the potential utility of DCM as a “mathematical microscope” that can probe synaptic quantities from the distant perspective of noninvasive electrophysiological data. Our biophysically interpretable DCM quantifies synaptic signaling at excitatory (glutamatergic) synapses with both fast AMPA and slow nonlinear NMDA receptors, and at inhibitory synapses, employing fast GABA_A_ receptors. The ensuing neuronal population dynamics are characterized by differential equations describing the temporal evolution of membrane potentials and ion channel conductances that underpin field potentials, including those recorded by MEG [6].

### Working Memory under L-Dopa

**Figure 1:**
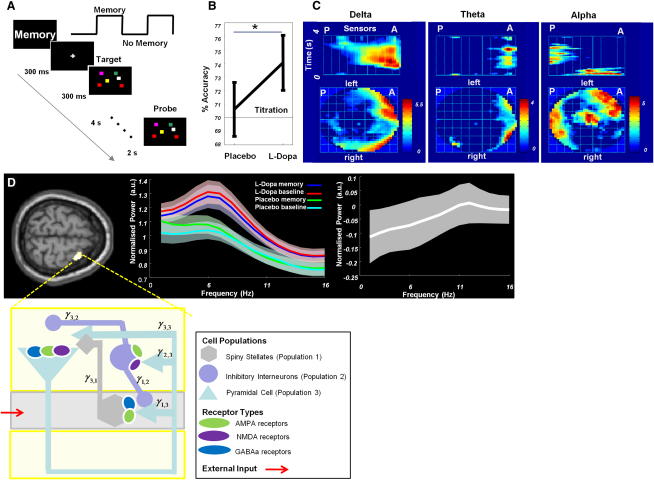
Experimental Task and Delay Period Activity (A) The experimental design comprised blocks of memory and no-memory trials. We ran six blocks of alternating memory and no-memory conditions, comprising 40 trials per block to yield a total of 240 trials per session. Drug and block order was counterbalanced across subjects. The first trial of each block was preceded by a cue indicating a memory or no-memory condition, and the background color was set to black for memory blocks and gray for no-memory blocks. Each trial consisted of a fixation cross (300 ± 50 ms) followed by the target stimulus of a colored square array. These arrays consisted of randomly colored squares (2.5° × 2.5°; red, blue, green, yellow, gray, cyan, and violet, with the number of squares corresponding to titrated load) in a randomized position. The target stimulus appeared for 300 ms and was followed by a delay period of 4000 ms, during which subjects had to retain the target memory array. This was followed by the onset of the probe image, after which subjects had 2000 ms to respond with a “match” or “no match” button press. Match and no-match trials occurred randomly, with equal probability. No-memory blocks contained the same stimuli, but subjects were instructed to simply press the “match” button on presentation of the second array. Accuracy was computed as the percentage of correct button press responses in memory blocks. Before drug administration on week 1, each subject was tested using 50 sample trials with varying memory load (two to six squares) to titrate accuracy levels. For each subject, the memory load with accuracy closest to 70% was used for the subsequent MEG experiment. (B) A pre-MEG test titrated individual memory load to achieve 70% accuracy. During MEG recordings, placebo-treated subjects performed with an accuracy close to titrated levels (70.60% ± 2.02% standard error of the mean [SEM]) and improved significantly on L-Dopa (74.04% ± 2.07%; ^∗^p < 0.05, one-tailed paired t test). (C) We obtained scalp-time statistical parametric maps (SPMs) by testing for increases in sustained activity at particular bands during memory retention (see Supplemental Experimental Procedures). Sustained increases in delta and theta responses were found over prefrontal sites, whereas sustained alpha occurred primarily over occipitoparietal sites. (Beta activity, although significantly greater at the beginning of the delay period, did not show a sustained effect during maintenance, and no effect was expressed in the gamma band. All other bands showed sustained increases.) The images are maximum-intensity projection images showing t statistics for a significance level of p < 0.01 for clusters with more than ten pixels; color bars denote t values. In the top panel, SPMs of the t statistic are depicted for all sensors (left to right, corresponding to posterior to anterior) over time. The bottom panels depict these same (largest) statistical values over time with corresponding sensor locations. (D) Left: SPM of the interaction between memory and dopamine (displayed at p < 0.01 uncorrected), where the peak is observed in right superior frontal gyrus (SFG; peak x = 32, y = 4, z = 68; t = 2.79, p = 0.006 uncorrected within a mask of inferior, middle, and superior frontal gyri). The SPM is rendered on a canonical structural MRI scan, displayed on a horizontal section at z = 66. We also tested for the orthogonal main effect of memory and found significant increases in activity for memory compared to no-memory trials in bilateral prefrontal cortex, maximal over right dorsolateral prefrontal cortex (peak x = 22, y = 52, z = 0; t = 3.95, p = 0.001 uncorrected within a mask of inferior, middle, and superior frontal gyri; Figure S3). Middle: average spectral density for memory and no-memory trials, on L-Dopa (blue and red lines, respectively) and on placebo (green and cyan, respectively), in the right SFG, averaged across subjects (shaded regions report the SEM). Right: the interaction ([memory L-Dopa − no-memory L-Dopa] − [memory placebo − no-memory placebo]) plotted from the spectra (middle panel) averaged across subjects shows a negative theta response when retaining a memory on L-Dopa relative to placebo. This is effectively the difference in the differences among the four condition-specific responses. Bottom: macrocolumnar architecture used to model right SFG responses in the DCM analysis. The model comprises three interconnected cell layers, where spiny stellate cells occupy granular layer IV (population 1) whereas inhibitory interneurons (population 2) and pyramidal cells (population 3) occupy supra- and infragranular layers. For clarity, neurons in the infragranular layer are omitted. Extrinsic (e.g., thalamic) input enters the granular layer and signals propagate throughout the macrocolumn via intrinsic coupling parameters γ_to,from_. The model's parameters are associated with particular ionotropic receptor types: AMPA and GABA_A_ at all cell types, and NMDA at pyramidal cells and inhibitory interneurons. These synaptic parameters γ represent lumped coupling parameters that quantify the collective effect of a number of biophysical processes such as receptor binding and transmitter reuptake. The modeled SFG response is assumed to arise most prominently (80%) from the pyramidal cells' depolarization due to their dendritic organization, with a 20% contribution from the membrane potentials of the inhibitory interneurons and stellate cells.

We recorded MEG signals from 18 participants performing a working memory (WM) task on two separate occasions, in a placebo-controlled randomized within-subject design involving administration of the dopamine precursor levodopa (L-Dopa). To assess working memory, we used a change-detection paradigm (Figure 1A). On placebo, subjects performed close to a psychophysically pretitrated level (70.60% ± 2.02% [standard error of the mean] correct responses). We predicted that L-Dopa administration (100 mg) would induce a behavioral improvement in WM. This was indeed observed, with a small but significant increase in overall WM accuracy (74.04% ± 2.07%; p < 0.05, one-tailed paired t test; Figure 1B).

### MEG Spectral Characteristics during Working Memory

To localize the neuronal correlate of this behavioral effect, we first examined the MEG signal profile during the maintenance period of the WM task. Specifically, we tested whether any of five frequency bands, delta (2–4 Hz), theta (4–8 Hz), alpha (8–16 Hz), beta (16–32 Hz), or gamma (32–60 Hz), exhibited sustained activity that was greater for memory than no memory conditions. A differential pattern was observed within delta, theta, and alpha bands (p < 0.001; Figure 1C) at predicted locations over prefrontal sensors. Having established the frequencies manifesting sustained effects, we source localized these frequencies (2–16 Hz) for each subject individually (for details, see Supplemental Experimental Procedures available online). To address the key question of whether, and how, WM-induced activity was modulated by L-Dopa, we examined the contrast of WM × drug interaction and observed significant effects in a focal area of the right superior frontal gyrus (SFG; peak x = 32, y = 4, z = 68; t = 2.79, p = 0.006; Figure 1D). This region exhibited prominent theta activity during memory maintenance, with spectra under L-Dopa exhibiting a higher amplitude peak at 6–8 Hz (Figure 1D). Our ensuing DCM analysis focused on the spectral responses in this region.

### Synaptic Assay Using DCM

In traditional delayed match-to-sample WM tasks, the delay period is accompanied by maintenance activity in prefrontal cortex thought to reflect reverberatory activity in pyramidal cell networks, which retain stimulus-related information for the period when the target is off screen. The synaptic dynamics of several different transmitter systems and receptor subtypes interact to support this sustained activity. Glutamatergic action at NMDA receptors is critical in maintaining recurrent reverberatory dynamics within the pyramidal cell network [7], because this nonlinear voltage-gated ion channel has a slow time constant providing a near constant synaptic drive [8]. Conversely, AMPA signaling induces fluctuations in cell assembly firing and a susceptibility to interference [8, 9]. Strong network inhibition has also been used to explain persistent activity associated with stimulus-selective attractors [10, 11]. Our critical analysis involved fitting a biophysically plausible DCM to SFG spectral responses to estimate synaptic parameters underpinning sustained (delay period) activity and how they are modulated by dopamine. Dopamine, particularly through its actions at D1 receptors [12, 13], modulates the balance of excitation and inhibition in the PFC via diffuse afferent projections from midbrain neurons [14] that stabilize persistent activity. This is attributed to two known effects of dopamine on PFC function during working memory. The first is an enhancement in the conductance of GABA_A_ [15] and NMDA [16, 17] channels, with the latter requiring some (optimal) level of excitation mediated by fast ionotropic (AMPA) receptors [18]. The second is an attenuation of postsynaptic responses of layer III pyramidal cells to exogenous glutamatergic inputs (from other cortical areas, or from thalamus via layer IV granular cells) [19], thus reducing the influence of remote sources on local circuit activity. It is these mechanisms that we hoped to access quantitatively via our parameter estimates.

In our model, excitatory spiny stellate cells in layer IV received extrinsic (cortical and thalamic) inputs in the form of passive exogenous currents. We constructed a layered columnar architecture with glutamatergic projections from the input layer IV to pyramidal cells occupying supra- and infragranular layers, with excitation mediated by both AMPA and NMDA receptors postsynaptically (Figure 1D). Sustained activity of these pyramidal cells arises from feed-forward processing via recurrent collaterals and reciprocal connections to the spiny stellate cells. Inhibition was provided by inhibitory interneurons occupying supra- and infragranular layers. These GABAergic neurons targeted ionotropic GABA_A_ receptors at pyramidal and stellate cells and in turn received glutamatergic inputs from pyramidal cells via NMDA and AMPA receptors (Figure 1D). Our modeling approach is summarized in Supplemental Experimental Procedures, and all details concerning the mathematical properties of our model, its physiological plausibility, and statistical procedures for fitting can be found in previous methodological studies [6, 20–22].

**Figure 2:**
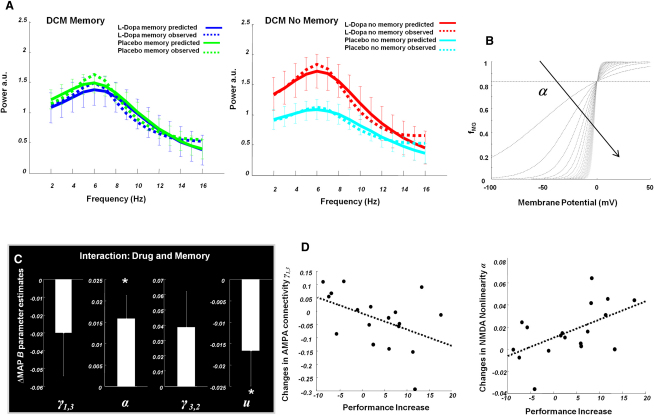
Dynamic Causal Modeling Predictions and Parameter Estimates (A) Predicted and observed spectral responses from a single subject. Left: the responses of the memory dynamic causal modeling (DCM) showing predicted spectral responses with 90% Bayesian credible intervals for both L-Dopa and placebo conditions. These credible intervals include the observed spectrum at all frequencies. Right: observed and predicted spectral responses (again with 90% credible intervals) for no-memory trials. Again, the credible intervals of our model predictions include the observed spectra. (B) NMDA nonlinear function (Equation 2, Supplemental Experimental Procedures) illustrated for increasing values of parameter α. As α increases, the voltage-dependent magnesium switch becomes highly nonlinear. (C) Synaptic measures illustrating the difference in maximum a posteriori (MAP) B parameter estimates from the memory and no-memory DCMs. Significant differences were tested by one-tailed t test in hypothesized directions (p < 0.05) and were observed for two parameters, reflecting an increase in NMDA nonlinearity α and decreased exogenous (glutamatergic) input *u* under L-Dopa versus placebo, while subjects engaged in working memory relative to a control condition. (D) Left: using the difference in MAP B parameter estimates, individual differences in AMPA coupling γ_1,3_ show a significant negative correlation with behavioral improvement on L-Dopa (R = −0.46, p = 0.027, Pearson one-tailed linear correlation). Right: the interaction in NMDA nonlinearity α shows a significant positive correlation with behavioral improvement on L-Dopa (R = 0.55, p = 0.009, Pearson one-tailed linear correlation).

To uncover the synaptic mechanisms underlying the observed drug × memory interaction, we inverted (fitted) two DCMs (memory and no memory) using the SFG spectral data from each subject. Condition-specific effects, reflecting differences in L-Dopa and placebo-induced processing, were modeled via a modulation of synaptic parameters (see Figure 2C and Supplemental Experimental Procedures for details), including the strengths of presynaptic inputs to and postsynaptic conductances of (1) AMPA and NMDA receptors at pyramidal cells and inhibitory interneurons and (2) GABAergic receptors at pyramidal cells. Additionally, we modeled changes in parameters encoding (3) the nonlinearity α of NMDA receptors and (4) extrinsic (cortical and thalamic) input *u* to layer IV cells. The spectral data predicted by the model recapitulated the increased theta band activity on L-Dopa. Clearly, changes in several or all of the synaptic parameters could contribute to theta band differences. We quantified the sensitivity of theta responses to each parameter. Testing at the peak of the interaction (6 Hz) we showed that the only parameter with a differential contribution to theta under L-Dopa and placebo was the nonlinearity parameter associated with NMDA receptors. Importantly, across the 2–16 Hz frequency range, the sensitivity profile was a different shape for each parameter, meaning that they can differentially promote or suppress spectral power (see Figure S2).

Our key question was whether pharmacologically induced changes in model parameters depend on the psychological state (i.e., memory condition). Of particular interest were those parameters representing processes expected to be modulated by dopamine. These were the AMPA pyramidal-to-stellate coupling, γ_1,3_, NMDA nonlinearity α, GABAergic connection strength γ_3,2_, and extrinsic input parameter *u* (Figure 1D; Figure 2C). Hence, we tested for task-induced differences in the DCM parameters on dopamine, using a repeated-measures analysis of variance with task (memory versus no memory) and parameter (γ_1,3_, α, γ_3,2_, and *u*) as within-subject factors. Crucially, we could show that on dopamine, there was a task-dependent difference in parameter estimates (p = 0.009). Analysis of the full (placebo-controlled) drug × task interaction showed consistent differences for two of the model parameters of interest, both of which relate to glutamatergic transmission (Figure 2C). Testing in the direction of hypothesized change using a paired one-tailed t test, we observed that the increase in the sensitivity (nonlinearity) α of NMDA receptors induced by L-Dopa versus placebo was further enhanced during memory compared to no-memory trials (p = 0.006). In contrast, L-Dopa versus placebo decreased the parameter *u* encoding exogenous (glutamatergic) input to layer IV, and this difference was significantly more pronounced during memory versus no-memory trials (p = 0.03). Corresponding tests of the interaction for parameters controlling GABAergic connection strength (γ_3,2_; p = 0.06) and AMPA-mediated coupling from pyramidal to stellate cells (γ_1,3_; p > 0.1) were not significant.

### Correlation between Behavioral Performance and Synaptic Assay

A key test of the validity of our estimates is whether the synaptic changes inferred by DCM predict observed behavioral improvements under L-Dopa. Given the antagonistic roles of NMDA and AMPA receptors for enabling reverberatory activity during WM [8], where NMDA to AMPA ratios have been proposed to be crucial, we focused on parameter estimates related to these receptor types. We found significant correlations between the change in behavioral accuracy under L-Dopa and the degree by which L-Dopa both decreased AMPA coupling (R = −0.46, p = 0.03; Figure 2D) and increased NMDA nonlinearity (R = 0.55, p = 0.01; Figure 2D). Put simply, subjects whose memory performance improved most on L-Dopa had greater NMDA gating and decreased AMPA signaling. In terms of its spectral signature, this performance enhancement was significantly correlated with a decrease in theta power for memory versus no-memory trials for L-Dopa relative to placebo states (p < 0.036; Figure S4).

## Discussion

In this study, we employed a “minimum simple model approach” [23], describing a candidate subset of possible synaptic mechanisms that may be modulated by L-Dopa. These mechanisms, which included synaptic transmission via AMPA, NMDA, and GABA_A_ receptors and glutamatergic inputs to layer IV, were chosen because of their important roles in WM delay period activity, as documented in both electrophysiological [24, 25] and computational studies [26]. Other possible effects induced by L-Dopa, e.g., an interaction with serotonergic transmission [27, 28], were not considered, and the specificity of the assay will require further testing. The sensitivity of the assay, however, was revealed by specific task-selective changes in cortical excitability in terms of dopamine-dependent changes in synaptic processes. These changes were consistent with the predicted modulatory effects of dopamine [8, 9, 29–31].

DCM is a general framework for testing mechanistic hypotheses of how measured signals are generated and, in so doing, can accommodate models of different types. Here we employed a DCM that followed closely the principles of well-established models of working memory. Computational models of the effects of dopamine on working memory demonstrate that prefrontal neurons settle on high-activity attractor states during memory maintenance, and that this dynamic behavior is caused by increased currents at NMDA- and GABA_A_-associated channels and decreased currents at AMPA receptor-associated channels [10, 26, 30]. The neural mass model underpinning our DCM contains the same types of active channels and cell types (where we also include stellate cells in the PFC's layer IV [32]) and uses differential equations that are formally similar to the leaky integrate-and-fire models of Brunel and Wang [10] (with an identical nonlinearity at NMDA receptors). Moreover, we model the relative contribution of these channels in a similar way, using parameters that specify the impact of presynaptic inputs on postsynaptic responses mediated by specific channels (Figure S1). However, in our analyses, we must consider a measurement obtained from an ensemble of tens of thousands of neurons. DCM affords inference on microscopic states from macroscopic data by employing a mean-field approximation [33]. This approximation replaces the time-averaged discharge rate of individual stochastic neurons with a common time-dependent average population measure (see [22] for a full treatment of this mean-field approach).

Our estimates of L-Dopa effects on synaptic transmission via several specific receptors correspond nicely to established neurophysiological effects of dopamine during WM tasks (Figure 2C). In particular, our modeling results replicate the known effects of NMDA and AMPA receptor function on delay-period activity, where L-Dopa increases postsynaptic responses mediated by NMDA receptors and decreases AMPA receptor-mediated coupling between pyramidal cells and stellate cells. Moreover, we found that L-Dopa decreased the impact of exogenous input from remote sources during memory maintenance; this is likely to reflect a diminution of noisy input from outside the circuit [19]. If the L-Dopa-induced enhancement of WM that we observe is due to enhanced reverberatory activity in prefrontal circuits, one would expect to find a significant correlation between the magnitude of our parameter estimates and behavior. This is exactly what we found: across subjects, drug-induced changes in parameter estimates encoding the effects of NMDA and AMPA signaling were significantly correlated with individual behavioral performance (Figure 2D).

Our wider strategic goal was to provide a proof of principle that it is possible to link human behavior via neural circuit models to specific synaptic signaling mechanisms. Explaining a behavioral effect in terms of synaptic mechanisms within specific brain regions provides us with a novel noninvasive framework for quantifying hidden biological mechanisms underlying measured data. Our approach may have considerable potential, not only for understanding fundamental cognitive processes but also for unraveling pathophysiological mechanisms in psychiatric and neurological diseases.

## Experimental Procedures

### Subjects and Pharmacological Manipulation

Eighteen right-handed, healthy volunteers (9 female, 9 male, age 27 ± 8 years) were studied. Volunteers attended two sessions, exactly 1 week apart, where they were given either 100 mg of L-Dopa dissolved in fruit juice or a fruit juice placebo 1 hr prior to scanning. The experimental procedures were approved by the local ethics committee of University College London.

### Dynamic Causal Modeling

For our DCM analysis, we extracted estimates of responses during the delay period from right SFG (see Supplemental Experimental Procedures). Two DCMs of identical structure were inverted per subject: one model was fitted to memory trial data (on L-Dopa and placebo), and the other was fitted to no-memory trial data. The posterior densities obtained by model inversion (and drug-induced differences) were subsequently used for inference on parameters [34, 35], enabling us to quantify the likely neural mechanisms generating different spectra over the four conditions. Note that by fitting separate DCMs to the two memory conditions, we allowed all parameters to change; however, the effects of L-Dopa were modeled within each DCM, with changes in a small plausible set of parameters. The equations describing this model are given in Supplemental Experimental Procedures.

## Supplemental Information

Document S1. Four Figures and Supplemental Experimental Procedures
