# Supplementary material for: An In Vivo Assay of Synaptic Function Mediating Human Cognition
Source: Curr Biol. 2011 Aug 9;21(15):1320–5. doi: 10.1016/j.cub.2011.06.053 (PMC3153654; doi:10.1016/j.cub.2011.06.053)
Supplement: Document S1. Four Figures and Supplemental Experimental Procedures [file mmc1.pdf]

Current Biology, Volume *21*

Supplemental Information

An In Vivo Assay of Synaptic Function Mediating Human Cognition

Rosalyn J. Moran, Mkael Symmonds, Klaas E. Stephan, Karl J. Friston, and Raymond J. Dolan

Supplemental Inventory

Figure S1. Neural Mass Model Equations of Motion

Figure S2. Sensitivity Analysis

Figure S3. SPM of Main Effect of Memory

Figure S4. Correlation between Performance and Theta Band Power

Supplemental Experimental Procedures
- MEG Analysis
- Dynamic Causal Modeling

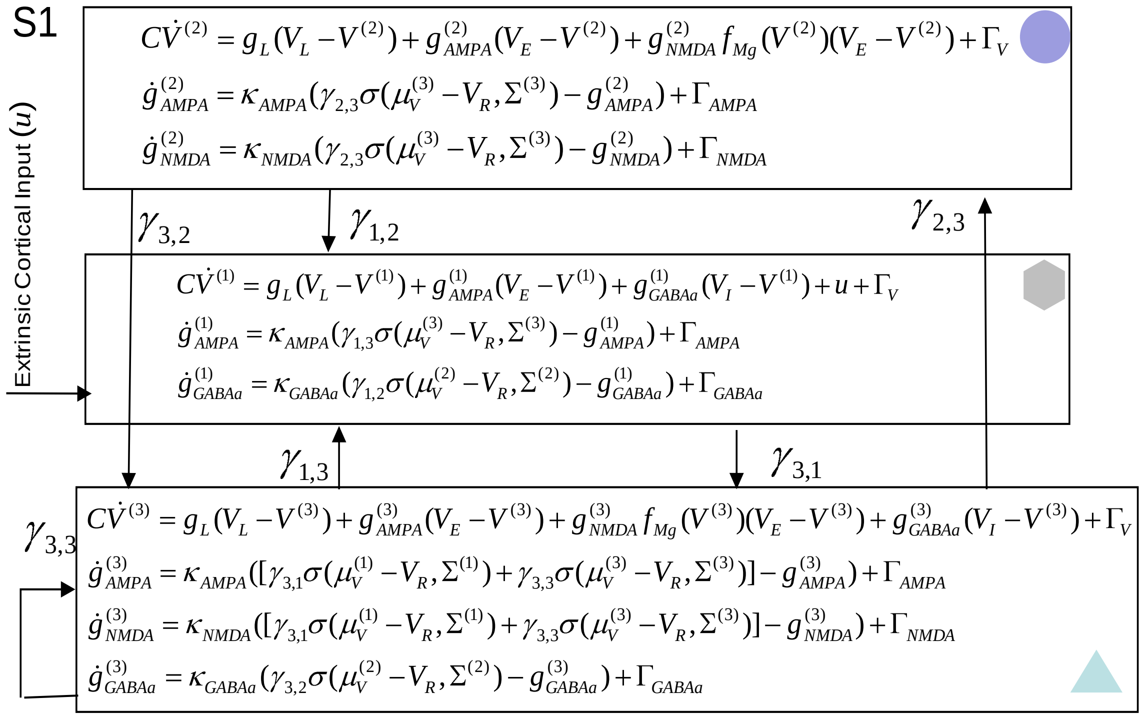

Figure S1. Neural Mass Model Equations of Motion

Morris Lecar-type differential equations (Moran et al. 2011) describing the time evolution of current and conductance at stellate cells (grey hexagon), inhibitory interneurons (purple circle) and pyramidal cells (green triangle). In this model, all cell types possess AMPA receptors, $GABA_A$ receptors are found at the stellate cells and pyramidal cells and NMDA receptors at pyramidal cells and inhibitory interneurons.

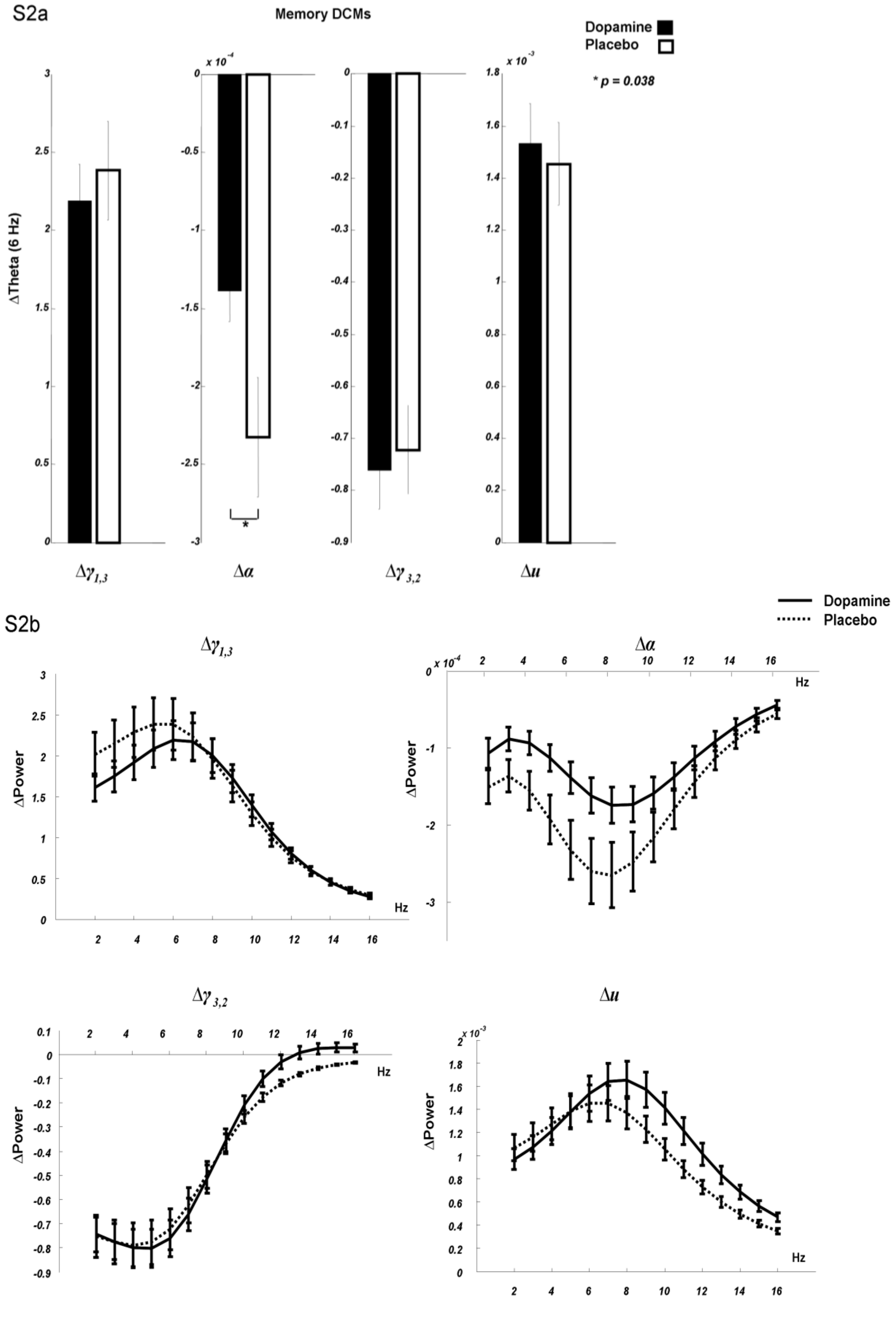

S2a
Memory DCMs
Dopamine
Placebo
* p = 0.038
ΔTheta (6 Hz)
x 10^-4
x 10^-3
Δγ1,3
Δα
Δγ3,2
Δu
S2b
Dopamine
Placebo
ΔPower
Hz

Figure S2. Sensitivity Analysis

(A) Here, we show the results of a contribution or sensitivity analysis, using the *a posteriori* estimates from the DCM of Memory trial data on and off L-Dopa from each subject. The results show the derivative of the predicted spectral response in the theta band with respect to the model's key biophysical parameters; i.e. the deviation of power in the theta band (at 6 Hz) with small deviations (increases) in parameters, [$\gamma_{1,3}$], [α], [$\gamma_{3,2}$] and [u] encoding AMPA responses, NMDA nonlinearity, GABA responses and exogenous input respectively. Note that two of these parameters dominate in controlling the power of the theta response. These are the AMPA parameter and GABA parameter, which enhance and diminish the power at 6Hz respectively. These derivatives are several orders of magnitude greater than those for the NMDA or input parameters. However the NMDA nonlinearity is the only parameter that contributes differentially under L-Dopa and placebo. At the optimum parameters under L-Dopa, changes in the α parameter lead to reduced diminution of theta, as compared to changes evaluated for the optimum parameters under the placebo condition (*$p = 0.038$ uncorrected; paired two-tailed t test over subjects).

(B) Different sensitivity profiles for each parameter across the fitted spectral range. A sensitivity analysis across all frequencies shows that during memory maintenance AMPA channels, [$\gamma_{1,3}$] differentially promote theta activity (peak at 5-6Hz), while small increases in the NMDA parameter], [α] also differentially effect theta/alpha bands (peak at 8Hz) but here the effect is to reduce the power at these frequencies. These AMPA effects are similar across the drug states, while the NMDA effect is greater for the L-Dopa network. A small increase in the GABA parameter, [$\gamma_{3,2}$] reduces power at all but the highest frequencies, whereby a qualitative change in sensitivity occurs at low beta frequencies (~13-16 Hz). Here GABA promotes the spectral power. This effect is more pronounced for placebo relative to L-Dopa architectures. An increase in input [u] also promotes power, preferentially in the alpha band (peak at 7-8Hz), where again this effect is higher in the placebo network.

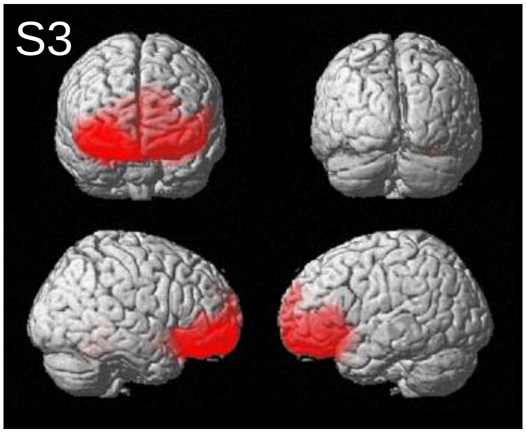

Figure S3. Main Effect of Memory

SPM of main effect of memory from source localized data rendered onto a canonical cortical surface, displayed at $p < 0.01$ uncorrected. Peak activity is observed in the right dorsolateral prefrontal cortex (peak $x = 22$, $y = 52$, $z = 0$; $t = 3.95$, $p = 0.001$ uncorrected within a mask comprising inferior, middle and superior frontal gyri).

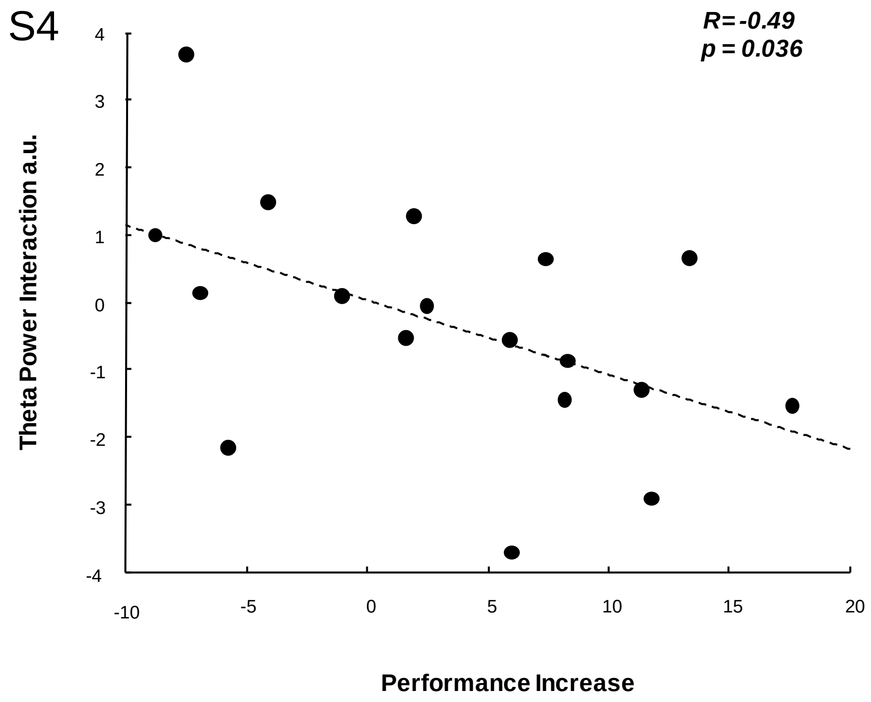

Figure S4. Correlation between Performance and Theta Band Power

Over subjects, individual performance increase on L-Dopa is inversely correlated with theta power (measured at the theta peak: 6Hz) for Memory relative to No Memory trials on drug relative to placebo.

Supplemental Experimental Procedures

MEG Analysis

Task and MEG Acquisition

MEG recordings were made in a magnetically shielded room using a 275-channel CTF system with SQUID-based axial gradiometers (VSM MedTech Ltd., Couquitlam, BC, Canada). The task involved a change-detection, visuo-spatial working memory task, where subjects retained a spatial array of colored squares [1] (see main text Figure 1A).

Data Preprocessing and Statistical Analysis

MEG data were epoched to obtain 4000 ms data segments corresponding to each trial's delay period. Data were corrected for artifacts using thresholding and then down-sampled to 200Hz (the analysis routines used for the present paper are available in the academic open source package SPM8; http://www.fil.ion.ucl.ac.uk/spm/).

SPM Analysis

Time-Frequency characterizations from the maintenance period for frequencies from 2-60Hz were rescaled logarithmically and averaged across memory and drug conditions resulting in 4 three-dimensional summary images per subject. These data were then converted to two-dimensional images by averaging within conventionally defined frequency bands, to produce band limited sensor × time representations at delta (2-4 Hz), theta (4 – 8 Hz), alpha (8 – 16Hz), beta (16 – 32 Hz) and gamma (33 - 60) frequencies. We computed a Memory – No Memory contrast image for each subject and entered these into a second-level (between subject) random effects SPM analysis using a one-sample t test. Using an uncorrected statistical threshold of $p < 0.01$ and a cluster size of ten pixels, we observed an increase in sustained activity at particular bands during memory retention (Figure 1C).

For our source space analysis, the inverse solution was calculated using a time window from 800 – 3200 ms during the maintenance period. SPM8's multiple sparse priors routine was used to estimate the cortical source of this activity [2]. A contrast of response power over 2 – 16Hz (the frequencies of interest obtained from the sensor level analysis) was computed for each memory and drug condition separately. Source activity measures were then interpolated into MNI voxel space [3]. We then tested for two effects of interest in source space, within an *a priori* region of interest, (comprising inferior, middle and superior frontal gyri) employing a random effects SPM analysis as above, using one sample t tests across subjects. This region was defined anatomically, using a mask image from the Wake Forest University PickAtlas [4]. After localizing these effects, we examined the effects of memory retention independent of the drug condition; using subject-specific contrast images for average [Memory – No Memory] conditions and the effects of drug on memory retention, to examine the interaction [$(\text{Memory} - \text{No Memory})_{\text{L-Dopa}}$ – $(\text{Memory} - \text{No Memory})_{\text{placebo}}$].

Source Extraction

For a smooth estimate of the delay period signal from SFG (centered on x = 32, y = 4, z = 68; MNI coordinates), we employed FieldTrip's beamforming source extraction method with regularization of 0.01% (http://www.ru.nl/neuroimaging/fieldtrip/). Frequency domain representations were constructed from this signal in the time window 800 – 3200 msec. These spectral responses, which were consistent with previous electrophysiological studies of working memory [5-7], were used for the inversion of our DCM.

Dynamic Causal Modeling

The principle of applying a mathematical model to infer the likely causes of measurements underpins many neurophysiological analyses, for example general linear or dynamic models that relate hemodynamic measurements [8] to underlying neuronal population activity. Dynamic Causal Modeling (DCM) is one such framework that employs Bayesian techniques to infer hidden (unobservable) neuronal mechanisms and architectures from neuroimaging or electrophysiological data [9].

Our mean-field model predicting neural activity in the SFG was based on the Morris-Lecar model [10]. The dynamics of postsynaptic responses are described in analogy to an equivalent RC circuit where, using Kirchhoff's current law, capacitive synaptic current flow equals the summed active and passive currents across the membrane [11,12]. The active currents across the postsynaptic membrane include ligand-gated excitatory (Na^+) and inhibitory (Cl^-) ion flow mediated by fast AMPA and $GABA_A$ receptors and slower NMDA receptors [22], depending on cell type. The magnesium block of NMDA receptors requires a sufficient transmembrane potential difference for its removal, and hence these channels are voltage-gated and non-linear. In our model, the NMDA conductance is augmented by a parameterized nonlinear sigmoid gain function $f_{MG}(V)$ (Eq. S1). Reversal potentials V_E, V_I and V_{NMDA}, were fixed at 60 mV, -90 mV and 60 mV, respectively, and membrane capacitance at 8 uF. A potassium leak current was used to account for all passive ionic currents, with reversal potential V_L of -70 mV. The model also included a driving current input *u*, entering at the granular layer:

$$C\dot{V} = \sum_{k \in L,E,I} g_k (V_k - V) + g_{NMDA} f_{MG}(V)(V_{NMDA} - V) + u + \Gamma_V$$
$$f_{MG}(V) = \frac{1}{1 + 0.2\exp(-\alpha_{NMDA} V)} \qquad \text{(S1)}$$

The conductances of the ligand-gated ion channels in our model are described by additional differential equations. These conductances *g* reflect the number of open channels, which are hidden states that depend on the presynaptic input ς, the coupling of presynaptic input to the postsynaptic response γ, the number of open channels and the channel's time-constant $(1/\kappa)$ with prior values of 4 ms, 16 ms and 100 ms, for AMPA, $GABA_A$ and NMDA receptors respectively.

$$\dot{g}_k = \kappa_k (\varsigma_k - g_k) + \Gamma_g$$
$$k \in \; I, E, NMDA \qquad \text{(S2)}$$

These dynamics at the single neuron level are stochastic, with the term Γ denoting Gaussian state-noise (fluctuations). As established by Marreiros et al [12], we can transform these single-neuron stochastic dynamics to a deterministic generative model of ensemble or population dynamics using the Fokker-Planck formalism. For the DCM, the prefrontal cortical region comprises three neuronal populations [13, 14], excitatory spiny stellate cells, pyramidal cells and inhibitory interneurons, coupled through intrinsic synaptic connections (see main text Figure 1D and Figure S1) with fast AMPA and $GABA_A$ receptors in all populations and NMDA receptors in pyramidal and inhibitory populations [15]. In a system of connected neuronal ensembles, the input to an ensemble *i* is the expected firing rate from the source ensemble *j*: The coupling of this presynaptic input to a postsynaptic response is represented by the parameter $\gamma_{i,j}$ which subsumes various biophysical processes such as receptor binding and transmitter reuptake.

$$\varsigma = \gamma_{i,j} \sigma(\mu_v^j - V_R, \Sigma^j) \qquad \text{(S3)}$$

The sigmoid function $\sigma(\cdot)$ represents the cumulative distribution function of the presynaptic depolarization density, $V = \mathcal{N}(\mu^{(j)}, \sum^{(j)})$, around a threshold potential V_R = -40mV, which determines the proportion of afferent cells firing (see Marreiros et al).

Given this set of differential equations we can apply their associated transfer functions in frequency space to the power spectrum of fluctuating inputs and predict the output spectrum. This allows us to establish a mapping from the system parameters to the predicted frequency spectrum [11]. Together with plausible assumptions about the form of the spectrum of neuronal fluctuations, this mapping furnishes a likelihood model that can be combined with prior densities on the parameters to yield a generative model of spectral responses (for details, see Moran et al. 2011). This model can be inverted (i.e.,, fitted to measured data; here the spectral density from the cortical source) to obtain the posterior densities of the parameters. In DCM, a variational Bayesian scheme (Variational Laplace) is used to approximate the posterior density over parameters by maximizing the negative free energy; a bound on the log-model evidence; see [16] for details. The parameters operate as scaling values on preset values [12]. Free parameters include gains on each channel's time constant $(1/\kappa)$, intrinsic coupling measures $\gamma_{i,j}$, extrinsic cortical input u, the NMDA channel nonlinearity parameter α and neuronal noise, represented by a mixture of white and pink noise components (parameter set *A*). Based on known neurophysiological effects of dopamine on different ion channels, a subset of these parameters was augmented by additional condition-specific scaling parameters to allow for drug-specific effects. These modulatory parameters (parameter set *B*) included changes in stellate to pyramidal cell coupling via AMPA and NMDA receptors ($\gamma_{3,2}$), pyramidal to pyramidal cell coupling via AMPA and NMDA receptors ($\gamma_{3,3}$), pyramidal to stellate cell connectivity via AMPA receptors only ($\gamma_{1,3}$), inhibitory interneuron to pyramidal cell coupling via $GABA_A$ receptors ($\gamma_{3,2}$), the NMDA nonlinearity (α) and extrinsic cortical input (u). The *B* parameters could take positive or negative values, representing an increase or decrease of the corresponding *A* parameters under L-Dopa. Prior densities on all parameters were found from a first pass of DCM for all subjects, where the posteriors from the best data-fit were used as priors in all subsequent analyses.

Supplemental References

1. Vogel, E., and Machizawa, M. (2004). Neural activity predicts individual differences in visual working memory capacity. Nature *428*, 748-751.
2. Friston, K., Harrison, L., Daunizeau, J., Kiebel, S., Phillips, C., Trujillo-Barreto, N., Henson, R., Flandin, G., and Mattout, J. (2008). Multiple sparse priors for the M/EEG inverse problem. NeuroImage *39*, 1104-1120.
3. Mattout, J., Henson, R., and Friston, K. (2007). Canonical source reconstruction for MEG. Computational Intelligence and Neuroscience *2007*, 17.
4. Maldjian, J., Laurienti, P., Kraft, R., and Burdette, J. (2003). An automated method for neuroanatomic and cytoarchitectonic atlas-based interrogation of fMRI data sets. NeuroImage *19*, 1233-1239.
5. Jensen, O., and Tesche, C.D. (2002). Frontal theta activity in humans increases with memory load in a working memory task. European Journal of Neuroscience *15*, 1395-1399.
6. Sauseng, P., Klimesch, W., Schabus, M., and Doppelmayr, M. (2005). Fronto-parietal EEG coherence in theta and upper alpha reflect central executive functions of working memory. International Journal of Psychophysiology *57*, 97-103.
7. Raghavachari, S., Kahana, M.J., Rizzuto, D.S., Caplan, J.B., Kirschen, M.P., Bourgeois, B., Madsen, J.R., and Lisman, J.E. (2001). Gating of human theta oscillations by a working memory task. The journal of Neuroscience *21*, 3175.
8. Buxton, R., Wong, E., and Frank, L. (1998). Dynamics of blood flow and oxygenation changes during brain activation: the balloon model. Magnetic Resonance in Medicine *39*, 855-864.
9. Moran, R., Stephan, K., Seidenbecher, T., Pape, H., Dolan, R., and Friston, K. (2009). Dynamic causal models of steady-state responses. NeuroImage *44*, 796-811.
10. Morris, C., and Lecar, H. (1981). Voltage oscillations in the barnacle giant muscle fiber. Biophysical journal *35*, 193-213.
11. Moran, R.J., Stephan, K.E., Dolan, R.J., and Friston, K.J. (2011). Consistent Spectral Predictors for Dynamic Causal Models of Steady State Responses. NeuroImage.
12. Marreiros, A., Kiebel, S., Daunizeau, J., Harrison, L., and Friston, K. (2009). Population dynamics under the Laplace assumption. NeuroImage *44*, 701-714.
13. David, O., Kiebel, S., Harrison, L., Mattout, J., Kilner, J., and Friston, K. (2006). Dynamic causal modeling of evoked responses in EEG and MEG. NeuroImage *30*, 1255-1272.
14. Fuster, J. (2008). The prefrontal cortex, (Academic Pr).
15. Homayoun, H., and Moghaddam, B. (2007). NMDA receptor hypofunction produces opposite effects on prefrontal cortex interneurons and pyramidal neurons. Journal of Neuroscience *27*, 11496.
16. Friston, K., Mattout, J., Trujillo-Barreto, N., Ashburner, J., and Penny, W. (2007). Variational free energy and the Laplace approximation. NeuroImage *34*, 220-234.
